# Supplementary material for: Friendship stability in adolescence is associated with ventral striatum responses to vicarious rewards
Source: Nat Commun. 2021 Jan 12;12:313. doi: 10.1038/s41467-020-20042-1 (PMC7804936; doi:10.1038/s41467-020-20042-1)

Friendship stability in adolescence is associated with ventral striatum responses to vicarious rewards

Schreuders et al. (2020)

Supplementary Information

Whole Brain Analysis: Winning versus Losing for Best Friend

We examined which brain regions showed significantly increased activation during winning > losing for a best friend with a whole brain analysis of variance (ANOVA) with three factors: feedback (2 levels: winning or losing for friend), type of friendship (2 levels: stable and unstable), and time point (3 levels: T1, T2, and T3). We first examined main effects of and interactions with feedback and friendship type, Family-wise error (FWE) correction, $p < .05$, $k \geq 10$. As expected, there was a main effect of feedback in the ventral striatum (MNI coordinates left: -9, 15 -3; right: 12, 15, -3) showing higher activity during winning than losing for the friend (Figure S1; Table S1; *unthresholded* data is also available for inspection here: https://identifiers.org/neurovault.collection:6024). The interactions with type of friendship and feedback were not significant at the whole brain level. Second, there was a main effect of time point; the results are displayed in Table S1. It should be noted that this whole brain analysis is not corrected for age (as this is not possible in whole brain ANOVAs). As SPM currently does not allow advanced longitudinal modeling, we proceeded with a ROI approach to investigate longitudinal effects and the role of friendship stability herein.

Context Effects of 'Other Trials' across three time points

The fMRI task also entailed trials for others (*disliked other* at T1 and *mother* at T2). To check whether these experimental conditions affected NAcc responses during winning and losing for friends and the self, we conducted a repeated measures analysis of variance (ANOVA) for the ROI-based values of left and right NAcc activity for Friend win > lose and Self win > lose. This resulted in four separate repeated measures ANOVAs (n = 103) with time point as within subject factor (3 levels: T1, T2, and T3), sex (2 levels: male and female)

and friendship stability (2 levels: stable and unstable) as between-subject factors; we also added age at T1 as covariate. There was no within-subject effects of time point on NAcc activity during winning vs. losing for friends (left NAcc: main effect of time point F (2, 97) = .18, p = .84, interaction effects with time point ps > .30; right NAcc: main effect of time point F (2, 97) = .31, p = .73, interaction effects with time point ps > .73), nor any between-subject effects of sex and friendship stability (ps > .30 and ps > .09 for left and right NAcc, respectively). Similarly, there was no within-subject effects of time point on NAcc activity during winning vs. losing for self (left NAcc: main effect of time point F (2, 97) = 1.82, p = .17, interaction effects with time point ps > .16; right NAcc: main effect of time point F (2, 97) = 2.15, p = .12, interaction effects with time point ps > .37), nor any between-subject effects of sex and friendship stability (ps > .26 and ps > .51 for left and right NAcc, respectively). Taken together, these analyses suggest that NAcc activity during winning and losing for friends and the self was not affected by changes in the trial procedure across the three time points.

Does Friendship Stability Modulate Age-Related Changes of Reward-Related Ventral Striatum Activity?

We used the same model fitting procedure as described in the manuscript to test which model best fitted left and right NAcc responses for the contrast between winning and losing for self, winning for friend and winning for self, and losing for friend and losing for self. The AIC and BIC values can be found in Table S2.

Neural responses in NAcc when winning > losing for self. We first tested whether sex explained additional variance beyond a model with a linear factor for age or a model with a quadratic predictor for age. Neither a main effect nor an interaction effect of sex explained additional variance (left NAcc: ps > .72.; right NAcc: ps >.96). Sex was therefore removed

from the model. We then tested whether friendship stability explained additional variance above the linear and quadratic predictors for age. Neither a main effect nor an interaction effect of friendship stability explained additional variance (left NAcc: *ps* >.25; right NAcc: *p*s >.47). The best fitting model for both the left and right NAcc was a model with a quadratic predictor for age (left NAcc: random effects: $SD_{\text{intercept}} = 0.34$, $SD_{\text{residual}} = 2.20$; fixed effects: [Intercept] $b = 1.94$, $SE = .15$, $p < .001$; [linear age] $b = -.01$, $SE = .04$ $p = .76$; [quadratic age] $b = -.02$, $SE = .01$, $p = .001$; right NAcc: random effects: $SD_{\text{intercept}} = 0.00$, $SD_{\text{residual}} = 2.40$; fixed effects: [Intercept] $b = 2.07$, SE =.16 , $p < .001$; [linear age] $b = -.04$, $SE = .04$ $p = .30$; [quadratic age] $b = -.03$, SE =.01 , $p < .001$).

Neural responses in NAcc when winning for friend > winning for self. We used the same model building procedure for left and right NAcc activity for the Friend win > Self win contrast. For left NAcc activity, sex did not explain additional variance to the model including linear and quadratic age terms (*p*s > .36), and neither did friendship stability (*p*s > .72). The linear and quadratic age terms were also not significant (*p*s> .26). For right NAcc activity, there were also no effects of sex and friendship stability (*p*s > .05, and > .78, respectively), nor of the linear and quadratic age terms (*p*s > .21).

Neural responses in NAcc when losing for friend > losing for self. We first extended the null model with a linear age term, and second with a quadratic age term. These steps showed the best fit for models including a linear age term (left NAcc: $p = .007$; right NAcc: $p = .03$, although for right NAcc only the AIC suggests a better fit above and beyond the null model). Above and beyond the linear and quadratic age terms, a main effect of sex or sex x age interaction effect did not improve the model fit (*p*s > .86 and > 91 for left and right NAcc respectively). Sex was therefore removed from the models. Extending the models with a main effect of friendship stability did also not improve the model fit ($p = .95$ and $p = .70$ for left and right NAcc, respectively). Although extending the models including a linear and

quadratic age term and a main effect of friendship stability with age x friendship stability interaction terms significantly improved the model fit for left NAcc (p = .03), the AIC and BIC fit indices show that the model fit did not improve above and beyond a model including only a linear age term (see Tabel S2). For right NAcc, extending the model with age x friendship stability interaction terms did not improve the model fit (p = .09). Therefore, for both left and right NAcc a model including a linear age term was selected as the best fitting model, (left NAcc: random effects: $SD_{\text{intercept}}$ = 0.00, SD_{residual} = 2.17; fixed effects: [Intercept] b = 0.61 , SE = 0.12 , p < .001 ; [linear age] b = -0.09, SE = 0.03, p = .008; right NAcc: random effects: $SD_{\text{intercept}}$ = 0.23 , SD_{residual} = 2.45; fixed effects: [Intercept] b = 0.56 , SE = 0.13 , p < .001; [linear age] b= -0.08, SE = 0.04 p =.03). These results show that across adolescence, NAcc activity related to losing for friends as compared to losing for self decreases with age (Figure S3 [A] for the fitted model, and [B] for the raw data).

Does Friendship Stability Modulate Age-Related Changes of Reward-Related Pleasure Ratings?

We used a model-building procedure to test which model best explained the pleasure ratings when playing for friends. The AIC and BIC values are reported in Table S3.

Pleasure from winning for friend. There was a linear decrease in pleasure from winning for friends with age (model fit p = .03, model summary fixed effects $b_{\text{intercept}}$ = 7.47, p < .001; $b_{\text{age,1}}$ = -0.06, p = .03; random effects: $SD_{\text{intercept}}$ = 0.93, SD_{residual} = 1.32). Friendship stability did not improve the model fit (ps > .56).

Pleasure from losing for friend. For pleasure from losing for friends, there was no effect of friendship stability. Although the model including friendship stability x age interactions fitted the data significantly better than a model including only a main effect of

friendship stability ($p = .02$), the fit indices (AIC and BIC) were not lower than those of the null model (see Table S3).

Correlations between Pleasure from Winning, Friendship Quality, and Closeness

Correlation analyses corrected for age were conducted to examine relations between pleasure from winning minus losing for friend, positive and negative friendship quality, and friendship closeness within time points (Table S4). At T1 positive and negative friendship quality correlated negatively ($p < .001$). There were no significant correlations at T1 for pleasure from winning and friendship quality (*p*s > .24). At T2, positive friendship quality correlated negatively with negative friendship quality ($p < .001$). Furthermore, friendship closeness correlated negatively with negative friendship quality ($p < .004$) and positively with positive friendship quality ($p < .001$). There were no significant correlations at T2 between pleasure from winning and friendship quality and friendship closeness (*p*s > .05). At T3, pleasure from winning correlated positively with positive friendship quality ($p = .007$) and friendship closeness ($p = .04$). Friendship closeness further correlated positively with positive friendship quality ($p < .001$). Correlations of negative friendship quality with pleasure from winning, and of negative friendship quality with positive friendship quality and friendship closeness were not significant (*p*s > .50).

Supplementary Table 1. Results whole brain 2 (feedback) x 2 (friendship type) x 3 (time point) ANOVA, FWE corrected, $p < .05$, $k \geq 10$, one-sided. L = left, R = right.

Brain Region	L/R	Voxels	z	*p* (FWE-corrected)	MNI coordinates		
					x	y	z
**Main effect of feedback**							
Ventral striatum	R	89	6.82	<.001	12	15	-3
	L	102	6.50	<.001	-9	15	-3
			5.83	<.001	-18	6	-9
**Main effect of time point**							
Occipital lobule	L/R	2470	Inf	<.001	24	-90	9
			Inf	<.001	-21	-93	9
			Inf	<.001	0	-84	0
Angular gyrus	R	218	Inf	<.001	36	-60	45
			7.84	<.001	45	-48	48
Lingual gyrus	L	53	6.91	<.001	-30	-45	-6
Inferior frontal gyrus	L	50	6.62	<.001	-36	36	-6
Superior frontal gyrus	L	101	6.58	<.001	-18	51	12
Middle frontal gyrus	R	58	6.52	<.001	48	27	42
			5.82	<.001	48	42	21
Supplementary motor area	L	98	6.46	<.001	-6	-3	54
			5.49	.001	0	9	51
			4.83	.023	0	6	63
Inferior frontal gyrus	L	23	6.12	<.001	-45	30	12
Hippocampus	R	28	6.12	<.001	32	-15	-21
Fusiform gyrus	L	18	6.00	<.001	30	-39	-12
Superior temporal gyrus	R	52	5.92	<.001	51	-27	0
			5.14	<.001	45	-36	6
Middle frontal gyrus	L	20	5.89	<.001	-51	24	39
			5.77	<.001	-57	18	36
Lingual gyrus	R	18	5.32	.002	15	-42	0
			5.17	.005	18	-51	3
Inferior parietal lobule	L	19	5.24	.003	-33	-57	45
		11	5.06	.008	9	30	33

Note. FWE correction, $p < .05$, $k \geq 10$

Supplementary Table 2. AIC and BIC values for NAcc activity to describe the relation with age, sex, and friendship stability. Preferred models are shown in bold. Df = degrees of freedom.

		Self win > lose			
		Left Nacc		Right NAcc	
	df	AIC	BIC	AIC	BIC
Null	3	1554	1566	1614	1625
+ Linear Age (1)	4	1554	1570	1611	1627
+ Quadratic Age (2)	5	**1546**	**1565**	**1599**	**1618**
+ Main effect Sex	6	1548	1571	1601	1624
+ Interaction Age and Sex	8	1552	1583	1605	1636
Age (1 and 2) + Main effect Friendship Stability[1]	6	1547	1570	1601	1624
+ Interaction Age (1 and 2) and Friendship Stability	8	1549	1580	1603	1634
		Friend win > Self win			
		Left Nacc		Right NAcc	
	df	AIC	BIC	AIC	BIC
Null	3	**1502**	**1514**	**1564**	**1575**
+ Linear Age (1)	4	1503	1518	1565	1581
+ Quadratic Age (2)	5	1505	1524	1566	1585
+ Main effect Sex	6	1506	1529	1564	1587
+ Interaction Age and Sex	8	1508	1539	1565	1596
Age (1 and 2) + Main effect Friendship Stability[1]	6	1507	1530	1568	1591
+ Interaction Age (1 and 2) and Friendship Stability	8	1511	1542	1571	1602
		Friend lose > Self lose			
		Left Nacc		Right NAcc	
	df	AIC	BIC	AIC	BIC
Null	3	1530	1542	1617	1628
+ Linear Age (1)	4	**1525**	**1540**	**1614**	**1629**
+ Quadratic Age (2)	5	1526	1545	1614	1633
+ Main effect Sex	6	1528	1551	1616	1639
+ Interaction Age and Sex	8	1532	1562	1620	1651
Age (1 and 2) + Main effect Friendship Stability[1]	6	1528	1551	1616	1639
+ Interaction Age (1 and 2) and Friendship Stability	8	1525	1556	1615	1646

[1]Sex did not improve the model fit and was removed from the model

Supplementary Table 3. AIC and BIC values for Pleasure from winning and losing for friend to describe the relation with age, sex, and friendship stability. Preferred models are shown in bold. Df = degrees of freedom.

		Pleasure from			
		winning		losing	
	df	AIC	BIC	AIC	BIC
Null	3	1355	1367	**1498**	**1510**
+ Linear Age (1)	4	**1352**	**1368**	1499	1515
+ Quadratic Age (2)	5	1354	1374	1499	1519
+ Main effect Sex	6	1355	1378	1498	1521
+ Interaction Age and Sex	8	1355	1386	1500	1531
Age (1 and 2) + Main effect Friendship Stability[1]	6	1356	1379	1501	1525
+ Interaction Age (1 and 2) and Friendship Stability	8	1359	1390	1498	1529

[1]Sex did not improve the model fit and was removed from the model

Supplementary Table 4. Correlation matrix of the self-report measures corrected for age. The table is showing Pearson's r correlations (two-tailed). Significant coefficients are in bold, *p < .05, **, p < .01, *** p < .001.

	Pleasure from winning (friend)	Positive friendship quality	Negative friendship quality
T1			
Pleasure from winning	-		
Positive friendship quality	.11	-	
Negative friendship quality	-.11	**-.36*****	-
Friendship closeness	n/a	n/a	n/a
T2			
Pleasure from winning	-		
Positive friendship quality	.18	-	
Negative friendship quality	-.08	**-.42*****	-
Friendship closeness	.07	**.50*****	**-.27**[1]**
T3			
Pleasure from winning	-		
Positive friendship quality	**.25**[2]**	-	
Negative friendship quality	-.02	.06	-
Friendship closeness	**.20**[3]*	**.53*****	.00

Notes. T = Time point. Pleasure from winning = pleasure from winning – losing for best friend. Friendship closeness at T1 is not available (n/a). [1]p = .004, [2]p = .007, [3]p = .035.

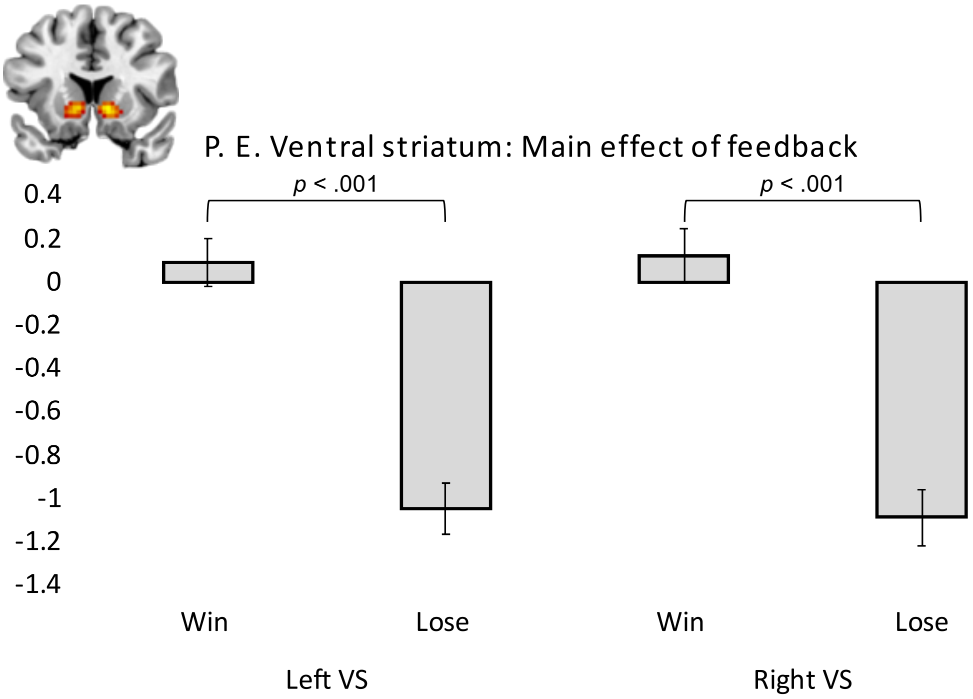

Supplementary Figure 1. **Visualization of the main effect of feedback Win > Lose when playing for friends.** Result were obtained with a 2 [win or lose] x 2 [stable or unstable best friendship] x 3 [T1, T2, or T3] whole brain ANOVA, FWE corrected, $p < .05$, $k \geq 10$, one-sided, Main effect of feedback yielded FWE corrected *p*s < .001. P.E. = Parameter estimates, VS = Ventral striatum.

Supplementary Figure 2. **Raw data of the age-related patterns and effects of sex and friendship.** Subpanels show age in relation to the following outcome variables. **A** left NAcc activity (*n* = 123, 346 data points), **B** right NAcc activity (*n* = 123, 346 data points), and **C** pleasure from winning (*n* = 123, 363 data points), **D** positive friendship quality (*n* = 123, 360 data points), **E** negative friendship quality (*n* = 123, 359 data points), and **F** friendship closeness (*n* = 122, 222 data points). Dots refer to data points, connected dots refer to one participant. If not specified in the subpanel, the legend explains the color features of the solid lines.

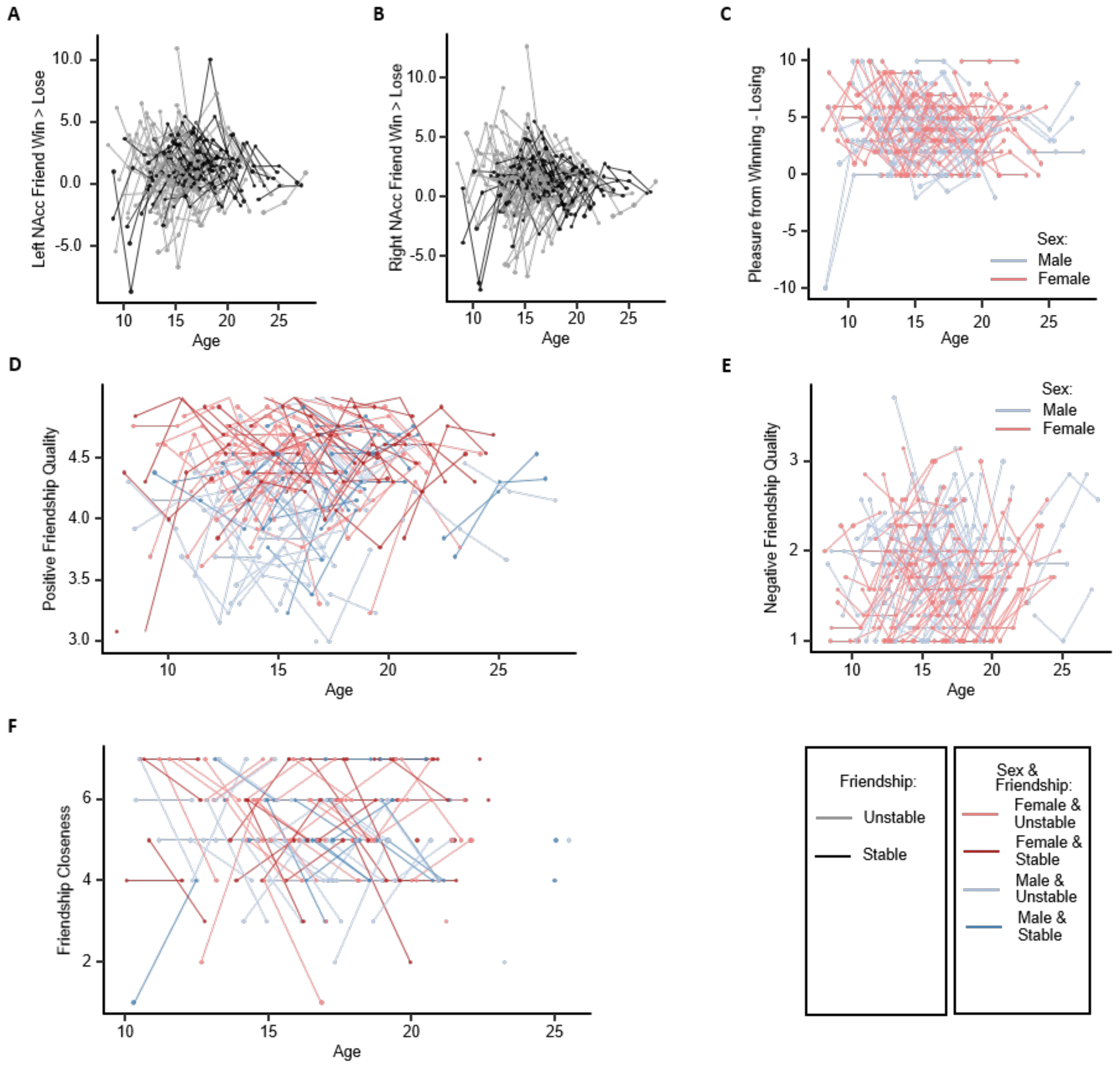

Supplementary Figure 3. **Relation between age and NAcc Friend lose > Self lose.** Subpanels show **A** the fitted mixed-model (*n* = 123, 346 data points) and **B** raw data. The grey ribbon shows the 95% confidence interval. The solid black line represents predicted values by the best fitting mixed-model.

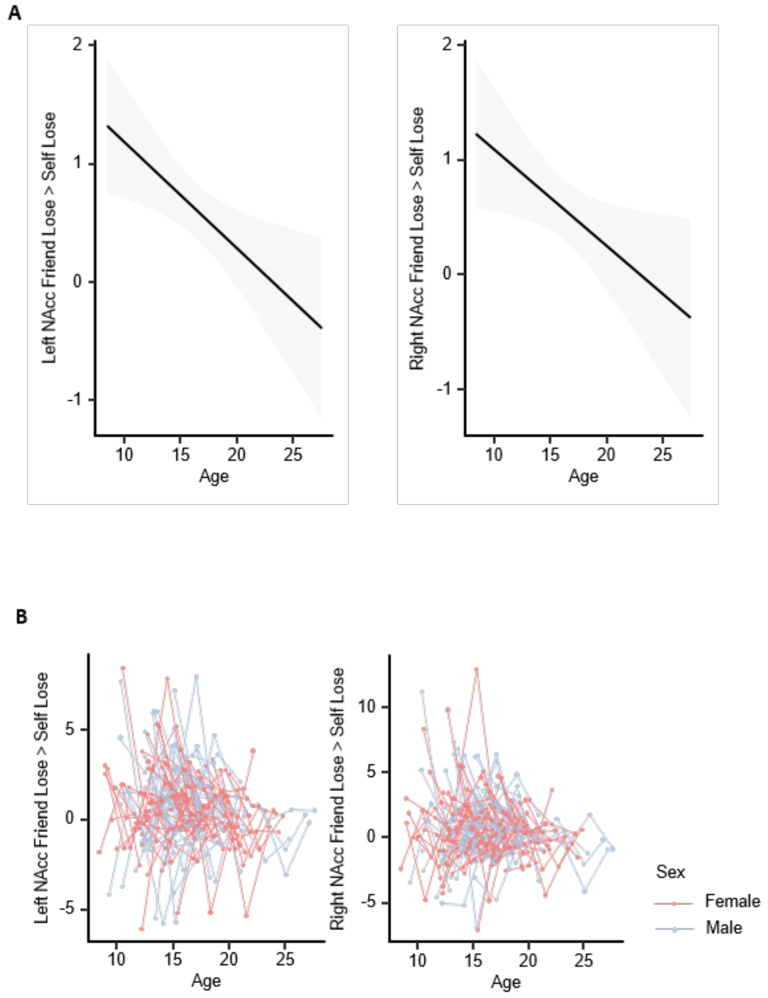

Supplementary Figure 4. Raw data of the relation between vicarious reward-related NAcc activity and friendship closeness in adolescents with unstable best friendships (*n* = 74, 135 data points) for **A** the left NAcc, and **B** right NAcc.

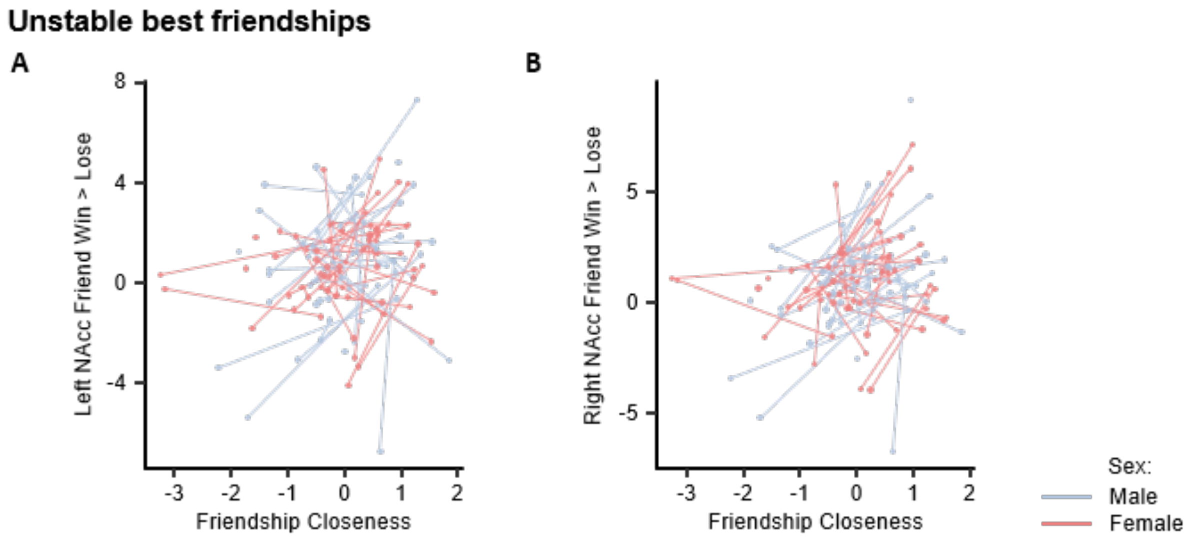

Supplement: Supplementary file 1 — Supplementary Information [file 41467_2020_20042_MOESM1_ESM.pdf]
