# Supplementary material for: Friendship stability in adolescence is associated with ventral striatum responses to vicarious rewards
Source: Nat Commun. 2021 Jan 12;12:313. doi: 10.1038/s41467-020-20042-1 (PMC7804936; doi:10.1038/s41467-020-20042-1)
Supplement: Supplementary file 3 — Reporting Summary [file 41467_2020_20042_MOESM3_ESM.pdf]

nature research

Corresponding author(s): Elisabeth Schreuders

Last updated by author(s): Sep 13, 2020

Reporting Summary

Nature Research wishes to improve the reproducibility of the work that we publish. This form provides structure for consistency and transparency in reporting. For further information on Nature Research policies, see Authors & Referees and the Editorial Policy Checklist.

Statistics

For all statistical analyses, confirm that the following items are present in the figure legend, table legend, main text, or Methods section.

n/a	Confirmed	
☐	☒	The exact sample size (*n*) for each experimental group/condition, given as a discrete number and unit of measurement
☐	☒	A statement on whether measurements were taken from distinct samples or whether the same sample was measured repeatedly
☐	☒	The statistical test(s) used AND whether they are one- or two-sided<br>*Only common tests should be described solely by name; describe more complex techniques in the Methods section.*
☒	☐	A description of all covariates tested
☐	☒	A description of any assumptions or corrections, such as tests of normality and adjustment for multiple comparisons
☐	☒	A full description of the statistical parameters including central tendency (e.g. means) or other basic estimates (e.g. regression coefficient) AND variation (e.g. standard deviation) or associated estimates of uncertainty (e.g. confidence intervals)
☐	☒	For null hypothesis testing, the test statistic (e.g. *F*, *t*, *r*) with confidence intervals, effect sizes, degrees of freedom and *P* value noted<br>*Give P values as exact values whenever suitable.*
☒	☐	For Bayesian analysis, information on the choice of priors and Markov chain Monte Carlo settings
☐	☒	For hierarchical and complex designs, identification of the appropriate level for tests and full reporting of outcomes
☐	☒	Estimates of effect sizes (e.g. Cohen's *d*, Pearson's *r*), indicating how they were calculated

Our web collection on statistics for biologists contains articles on many of the points above.

Software and code

Policy information about availability of computer code

Data collection	E-prime 2.0.10, 3T Philips Achieva MRI scanner
Data analysis	SPM 8, Rstudio 0.99.903, R version 4.0.2 nlme

For manuscripts utilizing custom algorithms or software that are central to the research but not yet described in published literature, software must be made available to editors/reviewers. We strongly encourage code deposition in a community repository (e.g. GitHub). See the Nature Research guidelines for submitting code & software for further information.

Data

Policy information about availability of data

All manuscripts must include a data availability statement. This statement should provide the following information, where applicable:
- Accession codes, unique identifiers, or web links for publicly available datasets
- A list of figures that have associated raw data
- A description of any restrictions on data availability

TO DO

Field-specific reporting

Please select the one below that is the best fit for your research. If you are not sure, read the appropriate sections before making your selection.

☐ Life sciences ☒ Behavioural & social sciences ☐ Ecological, evolutionary & environmental sciences

For a reference copy of the document with all sections, see nature.com/documents/nr-reporting-summary-flat.pdf

Behavioural & social sciences study design

All studies must disclose on these points even when the disclosure is negative.

Study description	We conducted a three-wave biannual functional magnetic resonance imaging study. Participants played an experimental guessing task in the scanner during which participants they win and lost money for their best friend. Hence, with the current study we obtained quantitative empirical data.
Research sample	To examine development across adolescence, participants between the ages of 8-28 years were included in the study. We collected data from 298 healthy, right-handed participants at the first time point (T1), 287 participants at the second time point (T2) and 274 participants at the third time point (T3), resulting in 205 participants that were included in each wave. From this sample, we identified two groups of participants based on their self-report of friendships over time: (a) individuals with a stable best friendship (n = 48), and (b) individuals with an unstable best friendship (n = 75). Sex was evenly distributed in both the stable and unstable friendship group: there were 28 females with stable friendships (58.3%) and 40 females with unstable friendships (53.3%). Participants were recruited in the area of Leiden University, The Netherlands. A convenience sample of typically developing participants was used.
Sampling strategy	The sample size was determined based on the grant proposal that was funded through the European Research Council- Innovative Ideas Program. Consistent with the ERC rules, there were no deviations from the proposed sample size. No formal power analyses were performed to determine the sample size, because of the longitudinal nature of the study.
Data collection	To assess nucleus accumbens activity, functional scans were acquired using a 3T Philips Achieva MRI scanner while participants played a heads-or-tails gambling game in which they had to guess which side of a coin would be chosen by the computer by pressing a button with their right index or middle finger. Chances of winning on each trial were 50%. The participants started the game with 10 coins. If they guessed correctly they earned more coins and if they guessed incorrectly they lost coins. The task was programmed in E-prime 2.0. A trial started with a screen showing how many coins could be won or lost (4000 ms) followed by a fixation screen (1000 ms). Next, participants were shown a feedback screen, which revealed whether they won or lost coins (1500 ms). The trial ended with a jittered fixation screen (1000–13200 ms). At time point 1 and time point 2, participants played 30 trials for themselves, 30 trials for their best friend, and 30 trials for another person (disliked peer at time point 1 and mother at time point 2). At time point 3, participants played 23 trials for themselves, and 22 trials for their best friend. To assess pleasure from winning, friendship quality, and friendship closeness, participants filled out online questionnaires that were entered in Qualtrics.<br><br>All participants underwent the same procedure (i.e., there were no experimental conditions that would require blind data collection). During testing, only experimenters and participants were present. Hypotheses were not formulated yet during data collection.
Timing	The current study entails three waves of data collection. The first wave of data collection was conducted in 2011-12, the second in 2013-14, and the third in 2015-16.
Data exclusions	We collected data from 298 healthy, right-handed participants at the first time point (T1), 287 participants at the second time point (T2) and 274 participants at the third time point (T3), resulting in 205 participants that were included in each wave. For the current study, we constructed two groups of participants based on participants' self-report of friendships over time: 48 participants with a stable best friendship and participants with 75 unstable best friendships throughout the entire study. Exclusion criteria were established before data analysis. Participants with a stable best friendship reported having the same best friend at each time point, whereas participants with unstable best friendships reported having a different best friend at each time point. The remaining 82 participants could not be categorized in one of these categories and were thus excluded from further analyses.<br><br>From the 48 participants with stable best friendships, there were in total 135 valid scans that could be used for the analyses (41, 47, and 47 scans obtained at T1, T2, and T3, respectively). Most scans were lost due to excessive motion (motion cut-off > 3mm movement in any direction) by the participant (six at T2 and one at T3). At T1 one scan was excluded due to a hole in the functional mask and at T3 one scan was excluded due to technical problems with the fMRI task. From the 75 participants with unstable best friendships, there were in total 211 valid scans that could be used for the analyses (66, 72, and 73 scans obtained at T1, T2, and T3, respectively). Again, most scans were lost due to excessive motion of the participant during scanning: eight at T1, two at T2, and two at T3. One scan was lost due to technical difficulties with the fMRI-task at T1 and one scan was excluded due to artifacts at T2.
Non-participation	From the total sample of 298 at T1, there were 11 (3,6%) participants and 25 (8%) that dropped out from all parts of the study at T2 and T3 respectively. Reasons given for non-participation included a lost of interest in the study, no time to visit the lab, or moving (abroad).
Randomization	Groups were formed bottom up, based on participants' self-report of friendships over time. We constructed a group of participants with stable and unstable best friendships stable and unstable best friendships based on best friend nominations provided at each of the three time points. Participants with a stable best friendship reported having the same best friend at each time point, whereas participants with unstable best friendships reported having a different best friend at each time point.

Reporting for specific materials, systems and methods

We require information from authors about some types of materials, experimental systems and methods used in many studies. Here, indicate whether each material, system or method listed is relevant to your study. If you are not sure if a list item applies to your research, read the appropriate section before selecting a response.

Materials & experimental systems

n/a	Involved in the study
☒	☐ Antibodies
☒	☐ Eukaryotic cell lines
☒	☐ Palaeontology
☒	☐ Animals and other organisms
☐	☒ Human research participants
☒	☐ Clinical data

Methods

n/a	Involved in the study
☒	☐ ChIP-seq
☒	☐ Flow cytometry
☐	☒ MRI-based neuroimaging

Human research participants

Policy information about studies involving human research participants

Population characteristics

See above

Recruitment

Participants were recruited through a participant database, schools and local advertisements. Participation in the study was open to all interested potential participants. Participants were included if they were between 8-25 years of age at the first time point and were free of any MRI contraindications, and psychological and neurological disorders. We expect no potential biases to influence the results.

Ethics oversight

Medical ethics committee of the Leiden University Medical Center, The Netherlands

Note that full information on the approval of the study protocol must also be provided in the manuscript.

Magnetic resonance imaging

Experimental design

Design type

event-related design

Design specifications

Participants played a heads-or-tails guessing game (chances of winning were 50%). A trial started with a screen showing how many coins could be won or lost (4000 ms) followed by a fixation screen (1000 ms). Next, participants were shown a feedback screen, which revealed whether they won or lost coins (1500 ms). The trial ended with a jittered fixation screen (1000–13200 ms).

Three different types of trials were included in the task to keep the participants engaged: trials on which participants could (a) win 3 or lose 3 coins, (b) win 5 or lose 3 coins, and (c) win 2 or lose 5 coins. Furthermore, at T1 and T2, participants played 30 trials for themselves, 30 trials for their best friend, and 30 trials for another person (disliked peer at T1 and mother at T2). At T3, participants played 23 trials for themselves, and 22 trials for their best friend.

Behavioral performance measures

N/A. (Regressors were modeled as zero-duration events at feedback onset and convolved with a canonical hemodynamic response function.)

Acquisition

Imaging type(s)

functional

Field strength

3T

Sequence & imaging parameters

The scanning procedure included (a) a localizer scan, (b) Blood Oxygenation Level Dependent (BOLD) T2* weighted gradient echo planar images (TR = 2.2 s, TE = 30 ms, sequential acquisition, 38 slices of 2.75 mm, field of view (FOV) = 220 mm x 220 mm x 114.7 mm), and (c) an anatomical 3D T1-weighted image (TR = 9.754 ms, TE = 4.59 ms, 8° flip angle, 140 slices, 0.875 mm x 0.875 mm x 1.2 mm, and FOV = 224 mm x 168 mm x 177.3 mm). Two functional runs with 45 trials each were obtained at T1 and T2. At T3, one functional run was obtained in which all 45 trials were presented in the same run. The first two volumes of the functional runs were discarded to allow for equilibration of T1 saturation effects.

Area of acquisition

We used anatomical masks of the left and right NAcc from the Harvard-Oxford subcortical atlas, thresholded at 40%. These anatomical masks included 28 voxels for the left NAcc and 26 voxels for the right NAcc. The MarsBar toolbox was used to extract the parameter estimates of the left and right NAcc for our analyses.

Diffusion MRI ☐ Used ☒ Not used

Preprocessing

Preprocessing software

The data were analyzed using SPM8 software (http://www.fil.ion.ucl.ac.uk/spm/). Preprocessing steps of functional images included realignment, slice-time correction, and smoothing using a Gaussian kernel of 6 mm full-width at half maximum. Statistical analyses were performed using the general linear model in SPM8.

Normalization	Functional and structural images were spatially normalized to T1 templates.
Normalization template	Templates were based on the Montreal Neurological Institute 305 stereotactic space.
Noise and artifact removal	motion cut-off > 3mm movement in any direction
Volume censoring	No volume censoring methods were used. Participants were excluded based on >3 mm in any directions. Motion parameters were included as confound regressors in the analyses.

Statistical modeling & inference

Model type and settings	Regressors were modeled as zero-duration events at feedback onset and convolved with a canonical hemodynamic response function.
Effect(s) tested	Region of interest parameters were extracted and used for the analyses

Specify type of analysis: ☐ Whole brain ☐ ROI-based ☒ Both

Anatomical location(s)	We used anatomical masks of the left and right NAcc from the Harvard-Oxford subcortical atlas, thresholded at 40%. These anatomical masks included 28 voxels for the left NAcc and 26 voxels for the right NAcc. The MarsBar toolbox was used to extract the parameter estimates of the left and right NAcc for our analyses.
Statistic type for inference (See Eklund et al. 2016)	voxel wise corrections were used for all whole brain analyses
Correction	A voxel-wise threshold was used of p<.05 Family Wise Error corrected (FWE)

Models & analysis

n/a	Involved in the study
☒	☐ Functional and/or effective connectivity
☒	☐ Graph analysis
☒	☐ Multivariate modeling or predictive analysis
